# The systematic activation method as a nursing intervention in depressed elderly: a protocol for a multi – centre cluster randomized trial

**DOI:** 10.1186/1471-244X-12-144

**Published:** 2012-09-18

**Authors:** Frans Clignet, Berno van Meijel, Annemiek van Straten, Pim Cuijpers

**Affiliations:** 1 Department of Clinical Psychiatry, VU University, Van Der Boechorststraat 1, Amsterdam, the Netherlands; 2 EMGO Institute, VU University Medical Center, Van der Boechorststraat 7, Amsterdam, BT 1081, the Netherlands; 3 Altrecht, Institute for Mental Health Care, Old Age Psychiatry, Dolderseweg 164, 3734 BN, Den Dolder, the Netherlands; 4 Inholland University of Applied Sciences, Research Group Mental Health Nursing, De Boelenlaan 1109, Amsterdam, HV, 1081, the Netherlands; 5 Parnassia Bavo Groep, Institute for Mental Health Care, Monsterseweg 93, The Hague, RJ, 2553, the Netherlands; 6 Mood Disorders Centre, Stocker road EX 4 4PY, Exeter, United Kingdom

**Keywords:** Major depressive disorder, Elderly, Inpatient, Behavioral activation, Nursing

## Abstract

**Background:**

Depression in later life is a common mental disorder with a prevalence rate of between 3% and 35% for minor depression and approximately 2% for Major Depressive Disorder (MDD). The most common treatment modalities for MDD are antidepressant medication and psychological interventions. Recently, Behavioral Activation (BA) has gained renewed attention as an effective treatment modality in MDD. Although BA is considered an easy accessible intervention for both patients and health care workers (such as nurses), there is no research on the effectiveness of the intervention in inpatient depressed elderly.

The aim of study, described in the present proposal, is to examine the effects of BA when executed by nurses in an inpatient population of elderly persons with MDD.

**Methods/design:**

The study is designed as a multi-center cluster randomized controlled trial. BA, described as The Systematic Activation Method (SAM) will be compared with Treatment as Usual (TAU). We aim to include ten mental health care units in the Netherlands that will each participate as a control unit or an experimental unit. The patients will meet the following criteria: (1) a primary diagnosis of Major Depressive Disorder (MDD) according to the DSM-IV criteria; (2) 60 years or older; (3) able to read and write in Dutch; (4) have consented to participate via the informed consent procedure. Based on an effect size d = 0.7, we intend to include 51 participants per condition (n = 102). The SAM will be implemented within the experimental units as an adjunctive therapy to Treatment As Usual (TAU). All patients will be assessed at baseline, after eight weeks, and after six months. The primary outcome will be the level of depression measured by means of the Beck Depression Inventory (Dutch version). Other assessments will be activity level, mastery, costs, anxiety and quality of life.

**Discussion:**

To our knowledge this is the first study to test the effect of Behavioral Activation as a nursing intervention in an inpatient elderly population. This research has been approved by the medical research ethics committee for health-care settings in the Netherlands (No. NL26878.029.09) and is listed in the Dutch Trial Register (NTR No.1809).

## Background

Depression in later life is a common mental disorder. Prevalence rates for depressive symptoms range between 3% and 35%, for minor depression approximately 10% [1], and for Major Depressive Disorder (MDD) 2% [2]. The prognosis of MDD in later life is poor: in three quarters of cases, the disorder becomes chronic [3]. MDD has serious consequences for everyday life (e.g. withdrawal from social activities, neglect of one’s self-care), with a risk of increased health care consumption [4,5]. MDD has one of the largest disease burdens, comparable with other chronic diseases such as diabetes or COPD. About one third of patients with MDD will be referred to a mental health care facility (ambulatory or residential) [5]. The elderly are particularly at risk of developing persistent MDD because of their vulnerability to physical illnesses, which may contribute to the onset and persistence of MDD [6].

The most common treatment modalities for MDD are antidepressant medication and psychological interventions (or a combination thereof) [7]. Antidepressants seem to be efficacious in treating late-life depression, although the treatment outcomes may be less positive for the subpopulation of older elderly [8]. There are several psychotherapeutic options in depression treatment. Among adults in general, these different options are comparable in their effectiveness [9]. Recently, Behavioral Activation (BA) has gained renewed attention as an effective treatment modality in MDD. In BA, patients learn techniques to monitor their mood and daily activities and to gain insights into the connection between the two. The patients then learn how to develop a plan that increases the number of pleasant activities and positive interactions with their environment. A meta-analysis has demonstrated large effect sizes for BA interventions (d = 0.89) when compared to a waiting list condition [10]. Furthermore, direct comparisons between Cognitive Therapy (CT) and BA have demonstrated that the effectiveness of the two interventions is comparable [11,12]. Since BA seems to be more accessible for many patients than CT, BA might be a preferred treatment option. Another meta-analysis shows that, in general, psychotherapy seems to be as effective for older individuals as for younger adults [9]. However, this meta-analysis did not include studies focusing on severely depressed or hospitalized patients. In general, psychological treatments have been found to be less effective in outpatients with chronic depression [13], and possibly severe depression [14], although the evidence is not conclusive [15].

Inpatient treatment remains an important treatment option for patients who cannot safely stay in their own environment [16]. Many of these patients suffer from severe and chronic forms of depression, and effective treatment options are needed to improve their recovery and reduce their suffering. The number of studies on psychological treatment for inpatients is limited. Recently, we summarized those studies in a meta-analysis and demonstrated small but robust effects [17]. However, there was a considerable variation in treatment setting, content of treatment, number of sessions, and inclusion and exclusion criteria applied. Furthermore, the quality of most of the studies was not optimal.

To our knowledge, there is only one study in which BA is tested in an inpatient population [18]. In this study, a total of 25 inpatient depressed adults were allocated either to BA (N = 10) or to Supportive Psychotherapy (SP) (N = 15). Despite the small sample, the study demonstrated the effectiveness of BA, with an effect size of 0.73. It is noteworthy that in this study, BA was executed by clinicians who had Master’s degrees, although BA is supposed to be an intervention which requires no complex skills.

The aim of study described in the present proposal is to examine the effects of BA when executed by nurses (RNs) in an inpatient population of elderly people with MDD. In this study, BA takes the form of a brief behavioral course of treatment lasting seven weeks, known as the Systematic Activation Method (SAM).

## Methods/design

### Study design

**Figure 1:**
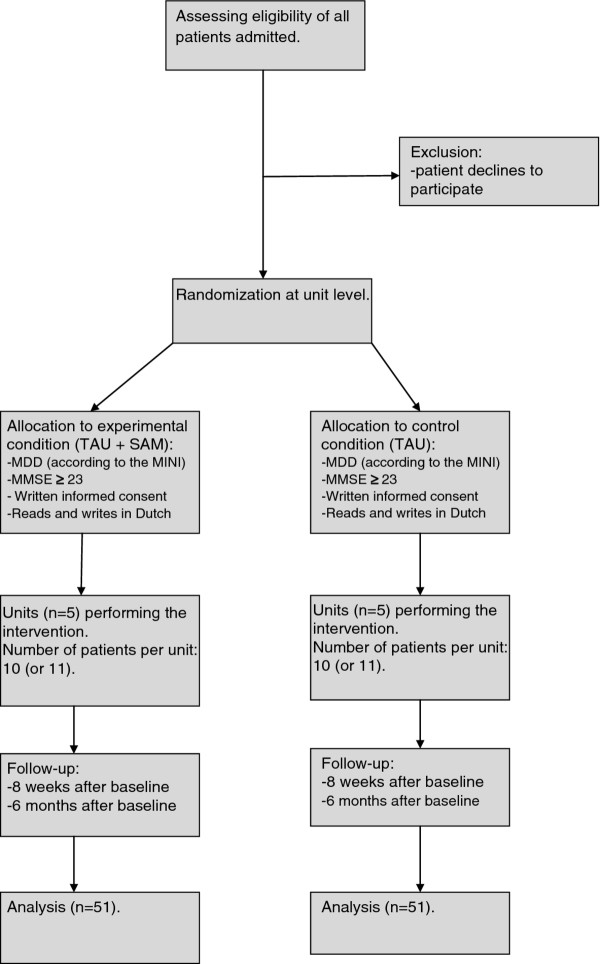
Flowchart.

The study is designed as a multi-center cluster randomized controlled trial with the participation of ten mental health-care facilities in the Netherlands. The Systematic Activation Method (SAM) will be compared with Treatment As Usual (TAU) for inpatient depressed elderly. In Figure 1 the study design is summerized.

The study has been approved by the medical research ethics committee for health-care settings in the Netherlands (No. NL26878.029.09) and is listed in the Dutch Trial Register (NTR No. 1809). 

### The units and randomization

The study will include ten mental health care units, which will be randomized to the experimental (SAM) or control (TAU) conditions. The following inclusion criteria will be used to select the units: (1) the units must specialize in the treatment of elderly patients with psychiatric disorders; (2) there must be at least three registered nurses (RNs) available who are able and willing to execute the intervention. Units that specialize in a specific disorder or treatment method (e.g. Electro Convulsion Therapy) will be excluded from the study.

Within each mental health institute, the aim is to find two units which are comparable. Matching of units is based on two criteria which will be applied in the following order: (1) level of restraint – both units should either be open or closed; (2) the presence of other treatments containing elements of the SAM, i.e. occupational therapy (because it focuses on activation), psychological treatment (because it focuses on influencing cognition).

For matching purposes, staff members at the participating units will be asked to fill in a self-developed evaluation form providing information on the specific features of the unit. This form includes a number of general questions concerning characteristics of the patient group, followed by more specific questions about the treatment program and level of restraint.

After matching the units into pairs, one unit will be allocated to the experimental condition and the other to the control condition. Allocation will be performed by an independent researcher (AvS) who will not maintain contact with the participating units. A random allocation generator will be used.

### Inclusion and exclusion criteria

The following patient inclusion criteria will be applied: (1) a primary diagnosis of Major Depressive Disorder (MDD) according to the DSM-IV criteria [19]. Patients with multiple diagnoses (e.g. comorbid personality disorder) are eligible as long as the primary diagnosis is MDD; (2) 60 years or older; (3) able to read and write in Dutch; (4) the patient must have consented to participation via the informed consent procedure.

Patients with severe cognitive problems (see below) will be excluded from this study because the intervention requires cognitive skills such as planning and evaluating activities and the structured monitoring of mood state.

### Recruitment of the study sample

The participating units will keep a list of all newly admitted patients and their primary diagnosis. All patients older than 60 with a primary diagnosis of MDD will be approached by a staff member and informed about the study. Once verbal consent is obtained and there are no cognitive problems according to the Minimal Mental State Examination (MMSE score ≥ 23) [20], the patients will be approached by one of the researchers. Confirmation of the psychiatric diagnosis will be executed by means of a diagnostic interview using the MINI Plus, a standardized instrument to assess psychiatric disorders according to the DSM-IV criteria [21]. Patients who satisfy the inclusion criteria will then be included in the study. Due to the nature of the intervention, it will not be possible to perform the study as either a single-blind or double-blind trial.

### Intervention

We developed the Systematic Activation Method (SAM) as a brief behavioral nursing intervention which focuses on increasing a positive mood change by increasing the number of positive activities. The SAM is based on Behavioral Activation (BA), developed by Lewinsohn and colleagues [22-27] and Hopko et al. [18]. The underlying assumption is that positive reinforcement of a low response rate acts as an eliciting stimulus for depressive behaviors and serves as a sufficient explanation for inactivity in a depressed individual [27]. The SAM is presented as a brief seven-week course. In order to make the SAM accessible for the elderly inpatient population, we have made some adjustments to the existing BA protocols. First, the goals of each session are described in the course book. This differs from the existing protocols, in which the first session is used for mutual goal setting. It is difficult to describe the overall goals of treatment for inpatient elderly with MDD; that is why the goals have been described at the beginning of each session. Second, the course is presented as a nursing intervention instead of a psychological intervention in order to increase its accessibility. Third, we simplified the activity logs to avoid overloading the patients. Fourth, the duration of the intervention has been shortened to seven weeks, in contrast to the existing treatment protocols, which last between eight and 15 weeks.

The SAM consists of six sequential steps: 1) monitoring the patient’s mood; 2) having the patient execute pleasant activities, randomly selected from an existing list of 49 activities [25]; 3) having the patient develop a positive activity plan; 4) having the patient explore how to use external resources; 5) setting up an activity experiment; 6) evaluation and consolidation. There is a one-week time interval for each step except the third step (developing a positive activity plan), for which two one-week intervals are required. If necessary, the time interval between the sessions can be reduced or extend. Each session is highly structured and starts with a review of the patient’s homework. After that, the nurse and patient discuss the central theme of the session and the patient is given his or her homework assignments for the coming week. The SAM is described in more detail by Clignet, Van Meijel, Van Straten, Lampe and Cuijpers [28].

The SAM requires the patient’s active involvement. This is often difficult due to the nature of MDD. The nurses participating in this study will therefore be given a brief training course of two four-hour sessions on guiding patients in executing the SAM. Training consists of two components. First, the nurses will be taught the structure and process of the SAM. Second, they will be trained in using motivational techniques. Many patients with MDD have difficulty engaging in structured activities, this being one of the essential components of the SAM. The use of motivational techniques is therefore vital for the effective execution of the SAM.

During the study, the participating nurses will receive training on the job, with the researcher visiting the units for in biweekly supervision meetings. In addition, the researcher will maintain telephone and e-mail contact. During the supervision meetings, the nurses will be invited to reflect on their experiences during execution of the SAM intervention and – in the event of problematic implementation – adjustments and alternative strategies can be discussed. The biweekly meetings will therefore be used to establish treatment integrity, along with a random audit of the course books and a written evaluation by the patients in order to register the received components of the SAM intervention.

The participating patients will receive the SAM as an individual adjunctive therapy combined with their existing primary treatment.

### Control group

The control group will receive Treatment As Usual (TAU). The most common treatment for patients with MDD is a combination of medication, occupational therapy and a form of psychological treatment such as Cognitive Behavioral Therapy (CBT) or Problem Solving Treatment (PST). Nursing care focuses on assisting patients in their self-care activities, and encouraging them to participate in the unit’s daily activity program. Nurses also discuss the patient’s overall progress on a regular basis (weekly or biweekly). TAU is recorded at unit level. Before participating in the study, the units describe their treatment program for MDD using a structured form developed by the authors. This form is based on the NICE standard for MDD [7] and the Dutch guideline for MDD [29].

### Assessment

All patients will be assessed at baseline, after eight weeks, and after six months. Each assessment will involve patients filling in a questionnaire. At baseline, we will collect demographic data (gender, social status, education, and ethnicity) and some information about the disease history (former episodes of MDD, frequency and nature of former treatments [outpatient treatments and/or clinical admittance], and psychiatric co-morbidity).

### Primary outcome

Our primary outcome is the level of depression. This is measured by means of the Beck Depression Inventory, second edition (BDI-II-NL) [30,31]. The BDI-II is a self-report scale which contains 21 items clustered in four response categories. The BDI-II is divided into two components, an affective component (e.g. mood) and a physical component (e.g. loss of appetite). The cut off scores are: 0 – 13 for minimal depression, 14 – 19 for mild depression, 20 – 28 for moderate depression, and 29 – 63 for severe depression. The Dutch version of the BDI-II has a high internal consistency (Chronbach’s α ≥ 0.90) and a strong correlation with other depression instruments [32].

### Secondary outcome

Secondary outcomes are: level of activity, anxiety, mastery, quality of life, costs and health care use.

An Activity Log (AL) will be used to measure the level of activity. This is a form in which the patient fills in his or her activities over the past week. The AL contains a week schedule which is divided into morning, afternoon and evening activities. The patients will be asked to fill in the activities they have executed during the past week at baseline and after six months. The number of activities and the type of activity will be used to calculate the activity level of each patient.

The seven anxiety items of the HADS (Hospital Anxiety Depression Scale) [33] will be used to measure anxiety. A four-point Likert scale is used to score the items (0 – 3) and the total score therefore ranges from 0 (no anxiety) to 21 (very anxious). The cut-off score is ≥ 8, as an indication for an Anxiety Disorder.

Mastery will be measured by means of the Pearlin Mastery Scale [34]. This is a five-item self-report scale measuring internal locus of control. The items are presented as statements to be scored on a five-point Likert scale. The scores on the Pearlin Mastery Scale range from 5 (minimum level of mastery) to 25 (maximum level of mastery).

Quality of life will be measured by means of the SF 36 (MOS Short Forms Health Survey) [35]. The scale contains 36 questions divided into eight subscales with three underlying dimensions: (1) Functional Status: physical functioning (10 questions), social functioning (2 questions), role functioning – physical problems (4 questions), role functioning – emotional problems (3 questions); (2) Welfare: mental health (5 questions), vitality (4 questions), pain (2 questions); (3) Evaluation of health care: general health perception (5 questions), change in health care (1 question). The response options vary from dichotomous to a six-point Likert scale. The SF-36 was translated into Dutch by Van der Zee & Sanderman [36].

Costs will be measured by means of the TiC-P (Trimbos/iMTA questionnaire for costs associated with Psychiatric Illness) [37]. This questionnaire consists of two parts: direct costs of care consumption and indirect costs of care consumption. The TiC-P is a broad questionnaire which can be adjusted to the relevant population. This study will make use of the categories ‘care consumption’, ‘informal care consumption’, and ‘use of medication’. All items have dichotomous ‘yes/no’ response options. The questionnaire will be completed by the patient as a self-report instrument.

Table 1 provides an overview of the instruments.

**Table 1:** Overview of instruments

**Time Instrument**	**Inclusion**	**T0**	**T1 = 8 weeks after T0**	**T2 = 6 months after T0**
Cognition: MMSE	X			
Diagnosis: MINI	X			
Baseline		X		
Depression: BDI-II-NL		X	X	X
Mastery: Pearlin Mastery Scale		X	X	X
Anxiety: HADS-A		X	X	X
Quality of life: SF-36		X	X	X
Health care costs: TiC-P		X		X
Activity Log		X		X

### Statistical analyses

The primary outcome is level of depression as measured with the BDI-II-NL. This outcome will be used to test the effect of the SAM as a nursing intervention in inpatient depressed elderly compared with TAU. The SAM is considered effective if there is a significant decrease in the level of depression in the treatment group compared to the control group. Because the patients will be randomized at unit level, multi-level analyses will be used to test the effects of the SAM intervention. In order to investigate differences in demographic and clinical variables, ANOVAs and Chi-square tests will be executed. Differences between the two groups in baseline characteristics will be corrected where necessary. For patient-oriented outcomes, Clinical Significant Change will be used [38,39].

Data will be analyzed according to the ‘intention-to-treat’ principle as well as the ‘completers only’ principle.

In order to correct for missing values, Last Observation Carried Forward and Multiple Imputation will be used as a sensitivity analysis.

Secondary outcome variables are quality of life (SF-36), costs (TiC-P), level of mastery (Pearlin Mastery Scale) and activities (Activity Log).

Anxiety is considered a co-variable because of its close association with MDD.

### Sample size

Effect sizes (d) will be used to calculate the sample size. A meta-analysis by Cuijpers et al. [10] found an overall effect size of d = 0.87 in favor of Behavioral Activation (BA) compared to a waiting list condition. A study in an inpatient population found an effect size of d = 0.73 in favor of BA compared to supportive therapy [18].

Our study will be performed in an inpatient elderly population with severe MDD. Because of the large effect sizes in previous studies, an effect size of d = 0.7 is expected. With α = 0.05 and a power (1-β) of 0.8, 34 patients are needed per condition. In other studies [10,40], the dropout rate shows a large variability (2% – 50%). For this study, we assume an average drop-out rate of 25%. Another rule of the thumb is to increase the study population by 25% when patients are randomized at unit level. That means that 51 patients are required for each condition. In total, 102 patients will be included in this study. The sample size was calculated using the G*power 3.0 software program [41].

## Discussion

This study will test Behavioral Activation (BA) as a nursing intervention in an inpatient elderly population. The study is innovative in two aspects. First, in previous studies psychologists with Master’s degrees carried out this intervention [10,11,18]. In our study, BA has been adapted to make it a nursing intervention – the SAM – to be carried out by registered nurses [28]. This makes BA more accessible for a larger group of patients.

Second, to our knowledge, most of the research on BA has mainly been conducted in outpatient adult populations. Only one other study [18] was executed in an inpatient adult population (N = 25) and, as far as we are aware, there are no studies in elderly inpatient populations. Studies in the elderly population are relatively scarce and have focused on outpatients [42,43] or depression in combination with dementia [44,45]. To our knowledge, this is the first BA effect study in an elderly inpatient population worldwide.

In addition to the innovative nature of this study, there are some difficulties concerning the study design. First, the study is vulnerable to selection bias. The SAM will be implemented in five mental health care units, and decisions regarding patient inclusion will depend partially on the anticipated efforts of the staff nurses. In order to minimize selection bias, the researcher will make an initial selection of eligible patients. Despite this, however, selection bias cannot be ruled out entirely, because it is ultimately the nurse who must motivate the patient to participate in the study, and this is expected to depend on the nurses’ belief that the SAM will be helpful for the patient. This may lead to selection bias in the experimental group, while the selection of patients in the control group will probably be free of bias. We will correct for any differences between the two groups in our statistical procedures.

This study will furthermore be vulnerable to information bias because a blind trial at intervention level is not possible. In order to avoid information bias, only self-report scales will be used in this study.

Finally, the standardized execution of the intervention is a point of concern. The SAM will be implemented at five units for elderly persons with psychiatric disorders. Although the SAM is presented as a highly prescriptive intervention, we expect that – due to differences in patient characteristics – individual variations in the execution of the SAM may occur that will be difficult for the researchers to control. Coaching meetings will be organized to promote treatment integrity.

### Ethical considerations

In the experimental condition, the SAM will be implemented as an adjunctive treatment modality. This means that patients with MDD will have an opportunity to benefit from the SAM while receiving treatment as usual. Although the study is intended for a broad category of patients, some patients will be excluded from the study, even though the nurses believe they could benefit from the SAM or parts thereof. These patients will also have an opportunity to engage in the SAM, but they will not be included in the study.

The SAM will be implemented in the control group units after all the patients have been included.

#### Trial status

The research is ongoing at the moment. We estimate that data-gathering will be completed in October 2012.

## Competing interests

The authors declare that they have no competing interests.

## Authors’ contributions

FC was responsible for the initial draft of this manuscript and for organizing and implementing the study. BvM, AvS and PC contributed to development of the SAM and the study design. BvM, AvS and PC also revised earlier versions of the manuscript. All authors read and approved the final manuscript.

## Pre-publication history

The pre-publication history for this paper can be accessed here:

http://www.biomedcentral.com/1471-244X/12/144/prepub
